# The importance of cross‐talk in research and the body: An early career researcher perspective

**DOI:** 10.1113/EP093955

**Published:** 2026-08-10

**Authors:** Lucy J. Ryan, Jamie A. Elliott, Robert Little, Mhairi A. Paul

**Affiliations:** ^1^ Institute for Neuroscience and Cardiovascular Research The University of Edinburgh Edinburgh UK; ^2^ United Kingdom Dementia Research Institute The University of Edinburgh Edinburgh UK

1

Interdisciplinary research is increasingly important for advancing science. Early‐career researchers (ECRs) play a central role in driving this progress and bridging gaps between fields. Inspired by a recent ECR‐focused multidisciplinary symposium, this editorial reflects on the importance of ECR engagement across disciplines and highlights examples of current interdisciplinary research.

## 2 THE IMPORTANCE OF INTERDISCIPLINARY RESEARCH FOR EARLY CAREER RESEARCHER DEVELOPMENT

Recent technological advancements have accelerated biological discovery and yielded unprecedented amounts of data (D'Argenio, 2018; Vitorino, 2024). However, this scientific progress has revealed ever‐growing complexity and elicited endless new questions. In recent decades, there has been an exponential growth in scientific publication (Bornmann et al., 2021; Pautasso, 2012) with modern scientific reports containing increasingly large datasets, diverse methodologies and requiring longer times to publish (Vale, 2015). While scientific discovery historically centred on the esteem of individual genius, the value of teamwork is undeniable in modern science. Evidence of the shift towards collaborative science is reflected in the increase in co‐authorship on publications (Vale, 2015; Zhou and Nunes Amaral, 2025). Indeed, there has been a sharp rise in the number of publications with 10 or more authors from the 1960s onwards (Zhou and Nunes Amaral, 2025), and leading journals such as *Cell* and *Nature* show a two‐ to four‐fold increase in authors per paper since the 1980s (Vale, 2015). Furthermore, publications authored by a team of researchers tend to be more highly cited than publications by solo authors, suggesting a higher scientific impact of collaborative studies (Wuchty et al., 2007).

Alongside the rise of collaborative science, interdisciplinary research has gained prominence. This is evidenced by research publications increasingly citing studies outside of their own discipline (Van Noorden, 2015) and high‐impact journals increasingly publishing interdisciplinary articles (Zhou and Nunes Amaral, 2025). Indeed, team diversity is associated with creativity and the emergence of new knowledge (Berliant and Fujita, 2008; Guimera et al., 2005; Pennington, 2016). Beyond improving the quality of science, interdisciplinary research advances professional development by challenging researchers to refine their ability to communicate concepts, become aware of the boundaries of their own discipline, stimulates learning, broadens perspectives and promotes innovative thinking (Mahringer et al., 2023). Moreover, interdisciplinary biomedical studies are more likely to be cited by patents, suggesting higher translational potential (Ke, 2023).

ECRs – comprising PhD students, post‐doctoral researchers and emerging principal investigators – constitute a large portion of the research community (Chisholm and Finelli, 2023; In support of early career researchers, 2021) with PhD students alone accounting for over 25% of the members within our own research institute. ECRs are integral to the research culture and increasingly value interdisciplinary research in their careers (Spence et al., 2024, 2026). ECRs are intimately involved with every stage of scientific research, from experimental design, data collection and analysis to interpretation and communication. Against the onerous backdrop of job insecurity and short‐term contracts, ECRs manage continual upskilling and adaptation in a fast‐moving, increasingly competitive research environment. Notably, these challenges disproportionally affect women who are more likely to face workplace hostility, career interruptions due to pregnancy and a disproportionate caregiving burden compared to men (Habicht et al., 2026; Jones and Floyd, 2024; Kwon, 2026; Spoon et al., 2023). Furthermore, researchers from under‐represented groups – including racial, sexual and gender identity, religious, disability‐based and socioeconomic minorities – face significant barriers in the academic workplace. Scholars from minority backgrounds experience social exclusion, receive fewer citations and hold fewer academic positions (Cech and Waidzunas, 2021; Hartmann, 2019; Kozlowski et al., 2024) with disparities compounded by intersectionality (Bertolero et al., 2020). Beyond a moral imperative, addressing the challenges faced by ECRs from under‐represented backgrounds is essential for the quality of science because, as for disciplinary plurality, demographic diversity is associated with scientific innovation (Hofstra et al., 2020). Often the scientists executing the experiments, ECRs show immense resilience by navigating failures and obstacles through creative problem solving. In addition, being relatively fresh in the field compared to established senior academics, ECRs bring recent knowledge and methodologies to the table as well as fresh perspectives that allow them to challenge assumptions and identify gaps in long‐standing dogma.

ECRs are under constant pressure to carve out their own research niche and therefore may feel reluctant to break away from tradition during this uncertain and busy career stage. Yet, in the rapidly evolving research landscape, it is clear that developing a narrow single‐disciplinary skillset is not sufficient to meet the demands of modern science. Though posing a potential cost to junior researchers in terms of time and resources, interdisciplinary research holds a number of benefits for ECRs including widening expertise, fostering creativity and motivation, increasing efficiency, building social and coordination skills, as well as expanding one's own network (Carroll et al., 2010; Mahringer et al., 2023; Pennington, 2016).

## 3 THE INSTITUTE FOR NEUROSCIENCE AND CARDIOVASCULAR RESEARCH (INCR) EARLY CAREER RESEARCHER DAY 2026

Recognising the benefits of and need for interdisciplinary connection, we recently organised a dedicated symposium for ECRs across the Institute for Neuroscience and Cardiovascular Research (INCR) at the University of Edinburgh. The institute was founded in August 2025 with the aim of fostering collaboration between neuroscience, cardiovascular, metabolic and psychiatric research, with researchers from these disciplines working within a shared environment. INCR is a multidisciplinary research institute at its core, recognising the benefit of integrating knowledge and approaches to tackle the complexity of whole‐body biological research across the lifespan. Encouraged and supported by the institute's leadership, we, a team of ECRs, organised the inaugural ‘INCR ECR day’ held on March 19^th^ 2026.

ECRs were the focus of this one‐day event, which aimed to spotlight their talent and the breadth of research within the institute while creating a relaxed space for ECRs to learn from each other and form new cross‐disciplinary connections. The event was attended by over 100 ECRs, not only from a wide variety of research disciplines but also from diverse career stages and cultural backgrounds. Female leadership and visibility were evident throughout the event; the organising committee was chaired by a woman and a high proportion of talented female ECRs contributed to the event as both presenters and session chairs. The day was opened by the institute's co‐directors, Professors David Wyllie and David Newby, who underscored the strengths of multidisciplinary research, which remained the guiding tone of the day. This was followed by an outstanding selection of ECR presentations – 8 flash talks and 10 extended research presentations – encompassing mental health, metabolism, neurodegeneration, neurodevelopment, cardiovascular and neurovascular disease. The interdisciplinary nature of the institute was clear as much of the research presented cut across these themes. It was inspiring to see the curiosity and engagement of ECRs from different research backgrounds, demonstrated through their insightful and thought‐provoking questions throughout the day. The day featured both a coffee and a lunch break during which 16 ECRs communicated their research through poster presentations, allowing for connection and knowledge exchange over refreshments. Attendees wore colour‐coded name tags indicating their primary research method to foster interdisciplinary conversations based on shared technical expertise. ECRs were informed about the institute's resources and support services with representatives sharing insights on innovation initiatives, funding opportunities, teaching experience, data management and core technical facilities.

The day closed with an inspiring keynote presentation from Professor Damian Bailey, Royal Society Wolfson Professor of Physiology and Director of the Neurovascular Research Laboratory at the University of South Wales. A true exemplar of interdisciplinary research with work focused on the integrated regulation of cardiovascular and neurovascular function, Professor Bailey demonstrated the value of challenging convention and transcending disciplinary boundaries. Scientific curiosity was stirred in the audience as Professor Bailey presented his ground‐breaking work on extreme‐environment models. ECRs were left inspired as Professor Bailey captured the ethos of INCR and encouraged junior researchers to reject siloed work, form collaborations, expand networks and embrace interdisciplinary research. These insights shaped the conversations throughout the final networking session of the day, where attendees gathered over refreshments to continue discussions.

## 4 EMERGING DISCIPLINARY INTERSECTIONS IN BIOLOGICAL RESEARCH

Organising the symposium was a deeply rewarding experience. The INCR ECR day enabled us to recognise and appreciate the cross‐disciplinary nature of our own research areas. To illustrate this further, we draw from our diverse research backgrounds to feature a selection of current topics that cross disciplines.

### 4.1 Neuroscience and microbiology: The gut microbiome influences behaviour

The brain was traditionally viewed as a ‘command centre’ for the body, exerting top‐down control over processes such as digestion. More recently, this perspective has shifted in light of evidence that peripheral systems have an active role in influencing central nervous system (CNS) function. Gut–CNS crosstalk has received particular attention for controlling behaviour, through both indirect influences and synaptic connectivity.

Since the 19th century, the gut microbiome has been hypothesised to influence mood and behaviour, with support for this emerging in recent decades (Miller, 2018). For example, Kawase et al. (2017) demonstrated that neurotransmitter metabolism in the brain requires normal gut microbiota: mice without a gut microbiome exhibited significant reductions in GABA, the primary inhibitory neurotransmitter, and glutamine, precursor to the excitatory neurotransmitter glutamate, across multiple brain regions. Although extreme – no humans fully lack a gut microbiome – this nevertheless demonstrates reliance of the CNS on gut health.

More recent studies have identified surprisingly direct control of behaviour by the gut. For example, Liu et al. (2025) characterised a previously unknown sensory system in the colon which modulates feeding behaviour in response to microbes, via communication with the CNS. Combining in vivo and in vitro methods, the authors demonstrated that neuropod cells in the gut epithelium detect bacterial flagellae, and inhibit feeding behaviour through synaptic connections to the vagus nerve like those in the CNS. Their work suggests that the body has a dedicated ‘sense’ for gut microbiota, to whose stimuli the brain responds as for any other sensory input. Studies have also highlighted the role of neuropod cell–CNS communication in influencing food preferences. Both humans and animals prefer sugars over non‐sugar sweeteners, and studies of mice lacking taste receptors have demonstrated that this effect persists without taste signalling (de Araujo et al., 2008). By silencing synaptic communication between neuropod cells and the vagus nerve, which abolished this sugar preference, Buchanan et al. (2022) demonstrated that they also detect nutrients, which influences feeding preferences. However, the gut controls more than feeding behaviour: Lai et al. (2024) demonstrated that the gut microbiome has lasting influences on mouse locomotor behaviours, again via the vagus nerve. Interestingly, while antibiotic treatment decreased locomotion, reintroduction of *Lactobacillus reuteri* to the gut, which has a wide range of reported health benefits, rescued this phenotype (Mu et al., 2018).

Together, these studies highlight a strong integration between the gut and CNS, influencing both neurochemistry and behaviour. This raises important questions about how changes to the microbiome may contribute to psychiatric disorders, which is a rapidly‐growing area of research.

### 4.2 Renal physiology and neuroscience: The importance of kidney–brain cross talk in regulating salt intake

Maintenance of appropriate body Na^+^ concentration ([Na^+^]), including body fluid homeostasis, is vital for mammalian terrestrial life. This is a strong example of coordinated function between organ systems and reciprocal signalling and cross‐talk.

Humans’ internal balance of Na^+^ can be regulated by both physiological and behavioural processes. Homeostatic mechanisms in response to water deficit include increased water reabsorption by the kidneys in response to antidiuretic hormone, vasopressin, secreted from the hypothalamus, whilst simultaneous neural outputs promote drinking behaviour (Matsuda, 2025). Further, a Na^+^ deficit can be countered by angiotensin II and aldosterone activity promoting renal Na^+^ reabsorption alongside an increased drive or appetite for dietary salt intake (Noda and Matsuda, 2022; Zhang et al., 2023). However, for most people worldwide, daily salt intake far exceeds their physiological need and is habitually greater than the World Health Organisation's (WHO) recommended upper threshold. Despite physiological homeostatic processes to remove excess Na^+^, accumulated intake can significantly elevate blood pressure (BP), particularly for those who display an exaggerated response to salt intake, termed salt‐sensitivity (Bailey and Dhaun, 2024).

Clinically, hypertension is now the leading cause of cardiovascular mortality and morbidity worldwide (Mills et al., 2020), with salt sensitivity being an independent risk factor for adverse outcome (Bailey and Dhaun, 2024). Despite availability of multiple treatment options, only about 20% of patients have well controlled BP (The Lancet, 2023), leading to calls for novel therapeutic strategies.

In preclinical salt‐dependent models of hypertension, overactivation of the sympathetic nervous system contributes to elevated BP (Kandlikar and Fink, 2011). Clinical trials have shown renal denervation reduces BP, notably in the most hypertensive of patients, though there remains debate about the effectiveness of such procedures (Cluett et al., 2024; Guber and Kirtane, 2022). Intriguingly, a recent study has reported that specific ablation of rat renal sensory afferent nerves, as opposed to total ablation of both afferent and efferent fibres, whilst not reducing salt‐dependent BP, promoted reduced drinking preference of salt supplemented water (Lauar et al., 2024). As such, a potential appetite for NaCl is attenuated, with an implication being that unplugging the brain from the kidney offers a route to reduce salt intake (Bailey, 2024). Importantly, further work is required in this area, especially towards identifying possible specific changes in brain regions related to renal afferent denervation.

Questions remain regarding the balance between physiology and behaviour towards our appetite for salt. Recent work from Lauar et al. (2024) tentatively proposes the kidney as a source not just target of sympathetic activity, whilst future collaborations between neuro‐ and cardiovascular scientists may lead to identifying new clinically important targets for salt‐sensitive individuals.

### 4.3 Cardiometabolism and inflammation: How metabolic dysregulation shapes the inflammatory response after myocardial infarction

Scientific progress increasingly relies on cross‐talk, not only within biological systems, but also between researchers across different disciplines. Myocardial infarction (MI) exemplifies this need. In the UK, over 100,000 people are admitted to hospital each year with MI (British Heart Foundation, 2025), and although reperfusion therapies have improved early survival, patients remain at high risk of complications, particularly heart failure (Butler et al., 2025). As adult cardiomyocytes cannot regenerate, the infarcted myocardium undergoes a tightly regulated repair process involving inflammation, proliferation and scar maturation. The trajectory of this inflammatory response is therefore critical to the quality of cardiac repair, with disruption or modulation of immune populations shown to significantly alter outcomes (Alshoubaki et al., 2024; Horckmans et al., 2017).

Within this framework, inflammation is governed by continuous biological cross‐talk between immune cells, cardiomyocytes, and systemic metabolism. Following MI, neutrophils are rapidly recruited to clear necrotic tissue, followed by pro‐inflammatory monocytes and macrophages, and later reparative macrophages and regulatory T cells that support resolution and scar formation (Geerkens et al., 2025). The balance between these phases determines whether healing proceeds effectively or progresses towards adverse remodelling.

This system becomes particularly vulnerable in obesity, a major cardiovascular risk factor affecting around one‐third of adults in Scotland (Scottish Government, 2025). Obesity is characterised by chronic low‐grade inflammation and metabolic inflexibility, where switching between lipid and glucose metabolism is impaired. In the myocardium, this limits adaptation during ischaemia, increases oxygen demand and reduces cardiac efficiency (Ketema and Lopaschuk, 2025), which in turn is closely linked to impaired inflammatory resolution.

Importantly, this metabolic dysfunction also affects immune cells. Effective post‐MI repair depends on immune cells dynamically switching metabolic programmes to support inflammatory or reparative states. In obesity, this flexibility is reduced, disrupting coordinated inflammatory progression and impairing tissue repair. This may contribute to the conflicting evidence in the ‘obesity paradox’, where some studies report improved outcomes in obese patients following MI (Baumann et al., 2016), while others show increased fibrosis and adverse remodelling (Gao et al., 2025; Yang et al., 2022).

Understanding post‐MI repair is therefore strengthened by cross‐disciplinary cross‐talk between cardiology, immunology and metabolism, enabling deeper mechanistic insight and ultimately striving to improve patient outcomes.

### 4.4 Neuroscience, metabolism and physiology: The impact of exercise on white matter health

A further example of cross‐discipline biology is the complex relationship between exercise and white matter health. CNS white matter consists of nerve fibres insulated in a lipid‐rich substance termed ‘myelin’ that enables fast and efficient neuronal activity (Fields, 2008). The white matter comprises over half of the human brain and its integrity is essential for CNS function (Fields, 2008).

Aerobic exercise, known to have neuroprotective effects (Vecchio et al., 2018), has been shown to increase white matter integrity, measured by magnetic resonance imaging (MRI), in both young and older adults (Maleki et al., 2022; Steventon et al., 2021; Svatkova et al., 2015; Voss et al., 2013). In line with this, individuals who engage in less physical activity show decreased white matter integrity (Svatkova et al., 2015) and signs of white matter damage (Moon et al., 2018).

However, there are conflicting reports on the impact of aerobic exercise on white matter integrity with one report showing older adults with reduced white matter integrity following an aerobic exercise intervention (Clark et al., 2019). Furthermore, individuals with exercise dependency – who tend to excessively exercise – show lower white matter integrity (Xie et al., 2025). Although, it is unclear whether individuals had reduced white matter integrity prior to developing exercise dependence. Nonetheless, these reports challenge the assumption that exercise is universally beneficial to brain health. There may be an upper limit to aerobic exercise intensity and duration, beyond which the impact could become damaging. Indeed, a recent study suggests that marathon runners undergo a period of myelin loss post‐run followed by myelin regeneration (Ramos‐Cabrer et al., 2025). An emerging mechanism that may drive exercise‐induced white matter loss is disrupted energy metabolism. As prolonged exercise results in depleted glycogen stores (Alghannam et al., 2021), the transient loss of white matter may be due to myelin itself being used as a source of energy through fatty acid metabolism (Asadollahi et al., 2024). The impact of myelin being used as an emergency energy reserve is unclear but there may be deleterious consequences during ageing as myelin shows a reduced capacity to regenerate from mid‐life (Franklin et al., 2024; Shields et al., 1999).

The neuroprotective effects of exercise may require a balance of potency and tolerability (Maleki et al., 2022), likely depending on prior level of fitness, age and gender. In order to define the optimal window of aerobic exercise for brain health, studies should include detailed participant profiling along with physiological measurements such as glucose levels, heart rate, respiratory measures and perceived level of exertion (Maleki et al., 2022). This work underscores the interconnected nature of neuroscience, energy metabolism and exercise physiology and highlights the need for interdisciplinary research to harness the neuroprotective effects of exercise.

## 5 CONCLUSIONS AND FUTURE OUTLOOK

These examples of research demonstrate the advancements achieved by cross‐disciplinary collaboration. Interdisciplinary research is essential both for addressing complex scientific questions and for developing ECR careers (Spence et al., 2024).

Notably, the benefits of interdisciplinary research, such as the generation of fresh ideas and increasing translational potential (Ke, 2023; Mahringer et al., 2023), are not restricted to ECRs but stand to benefit senior researchers throughout their career. Furthermore, lab heads have a responsibility to support and encourage ECRs in their interdisciplinary pursuits and can lead by example by valuing interdisciplinary research in their own lab group (Carroll et al., 2010; Spence et al., 2026). This could involve designing interdisciplinary research projects, initiating interdisciplinary collaboration and recruiting scientists from different disciplines into the team. Indeed, fostering multidisciplinary environments, such as within a research team, promotes the emergence of interdisciplinary research (Carayol and Thi, 2005). Strong mentorship from senior academics is also needed to help ECRs strike the balance between developing depth in their discipline and pursuing interdisciplinary expertise (Spence et al., 2026). Support from academic leadership for interdisciplinary research will empower the next generation of ECRs to pursue ambitious experiments, deepen learning and drive innovation.

We call on researchers to actively engage with interdisciplinary research. There are a number of accessible ways in which scientists can broaden their own perspective while simultaneously fostering an interdisciplinary research culture such as attending seminars on a different research topic, launching a seminar series that bridges disciplines, initiating social events that connect researchers from different institutes and, taking a similar approach to our event, organising multidisciplinary symposia. There are myriad strategies that could be further implemented to increase interdisciplinary research such as facilitating visits to other labs outside of one's own discipline to learn new techniques and approaches, setting up an interdisciplinary mentorship programme between ECRs and senior academics from different disciplines as well as the creation of exploratory pilot grants and PhD studentships with interdisciplinary aims at the core.

The cross‐disciplinary curiosity and engagement between ECRs at the INCR ECR day was inspiring and highlighted the interdisciplinary nature of our own research. We encourage scientists, across all career stages, to embrace interdisciplinary research and to create opportunities that foster cross‐disciplinary knowledge exchange. We look forward to the contributions that this generation of interdisciplinary ECRs will bring to science.

## AUTHOR CONTRIBUTIONS

Lucy J. Ryan developed the editorial content and structure. Lucy J. Ryan wrote the sections ‘The importance of interdisciplinary research for early career researcher development’, ‘The Institute for Neuroscience and Cardiovascular Research (INCR) Early Career Researcher Day 2026’, ‘Neuroscience, metabolism and physiology: The impact of exercise on white matter health’ and ‘Conclusions and future outlook’. Jamie A. Elliott developed the title and wrote the section ‘Neuroscience and microbiology: The gut microbiome influences behaviour’. Robert Little wrote the section ‘Renal physiology and neuroscience: The importance of kidney‐brain cross talk in regulating salt intake’. Mhairi A. Paul wrote the section ‘Cardiometabolism and inflammation: How metabolic dysregulation shapes the inflammatory response after myocardial infarction’. Lucy J. Ryan, Jamie A. Elliott, Robert Little, Mhairi A. Paul contributed to the organisation of the manuscript and proofread the text. All authors have read and approved the final version of this manuscript and agree to be accountable for all aspects of the work in ensuring that questions related to the accuracy or integrity of any part of the work are appropriately investigated and resolved. All persons designated as authors qualify for authorship, and all those who qualify for authorship are listed.

## CONFLICT OF INTEREST

None declared.

## FUNDING INFORMATION

JAE was funded by Medical Research Scotland (PHD‐50496‐2022). [Correction added on 4 September 2026, after first online publication: The Funding Information has been updated.]

### GENERATIVE AI STATEMENT

The authors used AI to generate synonyms to aid phrasing while writing and, in some cases, to proof‐read text. It was not used to generate sections of text, images or data.
